# Pathways to Suicide among Police in Rajasthan: Perceptions and Experiences of Police Personnel

**DOI:** 10.3390/ijerph20031812

**Published:** 2023-01-18

**Authors:** Anne Krayer, Seema Kulhari, Vimal Sharma, Catherine Robinson

**Affiliations:** 1 Centre for Mental Health and Society, Bangor University, Bangor LL57 3YH, UK; 2 Medical & Health Department, Government of Rajasthan, Rajasthan Police Academy, Jaipur 302016, India; 3 Personal and Social Services Research Unit, University of Manchester, Manchester M13 9QS, UK

**Keywords:** police suicide, police service, India, organisational factors, occupational factors

## Abstract

*Background*: Evidence regarding the experience and perceptions of police personnel with suicide in South Asia is limited. This study explored the lived experiences and perceptions of suicide among police personnel in an Indian state. The focus was on explanations of and reasons for suicide. *Methods*: We conducted 20 qualitative interviews in 2021 with police of different ranks, guided by a topic guide. The reflexive thematic analysis approach was supported by the use of NVivo 12, a qualitative software package. *Results*: We explore three intersecting key themes around suicide in the police force, including: (1) the stressful police environment; (2) expectations of mental strength; and (3) police image and help-seeking. We discuss the tensions between these themes and how to address the challenges of supporting police personnel. *Conclusions*: To support and improve police personnel’s mental well-being training and support are needed but also broader changes at the organisational level. These need to take social and historical factors into account. An increased level of suicide and mental health literacy will not only benefit the police force but also the general public, and it would be very timely with recent changes in the Indian mental health and suicide policy context.

## 1. Introduction

Research from around the world suggests that the police are among the occupations at the highest risk for suicide, although evidence from South Asia is sparse [1]. In addition, the importance of the police in Asia and the Pacific as a key stakeholder in suicide research and prevention has been pointed out as early as 2010 [2]. 

Estimating suicide rates for police personnel is challenging due to a number of factors, including inconsistencies in reporting and a likelihood of underreporting [3]. Earlier research suggested that casualty rates for Indian police officers are some of the highest in the world [4]. There is a lack of current suicide statistics available for police personnel in India. Nathanael [5] suggested that between 2013–15, over 940 police personnel killed themselves in India. A recent report [6] on the Indian police found that the force is understaffed and under resourced, while at the same time crime rates have risen, making it a stressful environment to work in. 

More recently, it has been argued that aiming to classify suicide rates for police personnel is unproductive and that a better understanding of risk factors and pathways to suicidal behaviours is essential for suicide prevention in the police force [3,7]. Krishnan and colleagues [3], in a systematic review, identified five risk factors that increase the likelihood of suicide, including “problematic substance use close to, or at the time of death; presence of depression and previous suicide attempts; differences in trauma response; exposure to excessive and prolonged job-related stress, including dissatisfaction; absence of a stable intimate relationship” (p. 939). Importantly, the effects of the risk factors are cumulative. None of the studies reviewed were from India, and only a few were from South Asia. 

In a ground-breaking approach, Hart and colleagues [8], working with Australian police, showed that the experience of well-being is linked to organisational and operational factors. They confirmed distinct negative and positive organisational and operational factors. Shift work and working with victims of crime are examples of operational factors; this includes demands that one would expect when working in the police. In contrast, organisational factors include more strategic and managerial issues such as bureaucracy, supervision, and resource allocation. Analysis showed that, contrary to previous assumptions [9,10], organisational, rather than operational pressures contributed to low well-being. In other words, although work itself may be difficult, it is the organisational context that determines levels of well-being. This was confirmed in a subsequent paper [11]. They also emphasised the importance of individual issues.

Singh and colleagues [12] measured very high self-reported stress levels among a sample of police personnel in India. They found that good social support and adaptive coping strategies reduced the adverse effects of work stress. A stressful police environment can lead to health problems, including increased use of alcohol or drugs and more frequent suicidal thoughts [4,13]. The importance of considering the role of occupational stress and mental health in uniformed police has recently been highlighted in a special issue of the Indian Police Journal [14]. Di Nota and colleagues [7], working with Canadian police officers, proposed that several factors need to come together over time for suicide to happen. A police officer experiences hopelessness (which could be due to a number of issues) and lacks meaningful relationships while at the same time having the impetus and capacity to put a plan into action. 

Overall, most research focuses on police forces in developed countries in the West, and the research is overwhelmingly quantitative. There is very little qualitative research with police personnel in India and South Asia on their perceptions around suicide and their support and training needs. Thus, this qualitative study explored the perceptions of police suicide in a sample of Rajasthan police personnel.

## 2. Materials and Methods

The data presented here draws on 20 interviews conducted with police personnel in Rajasthan, India, in 2021. The data set is part of the larger South Asia Self Harm Initiative (SASHI), a Global Challenges Research-funded project. 

### 2.1. Setting

India’s justice and mental health systems are heavily influenced by the British. The present form of Indian policing is mainly based on the Police Act of 1861, which was introduced by the British and based on British-style policing. The act outlined the main functions of the police as maintaining law and order, preventing crime, surveilling, and controlling citizens [15]. The aim was to control the population and suppress dissenting voices. It has been argued that as part of this colonial legacy, current-day policing in India is still more focused on protecting the government and elite individuals’ interests than promoting democratic principles [16]

India is a republic made up of 29 states and eight union territories (https://knowindia.india.gov.in/states-uts/, accessed on 6 December 2022). Although the Indian Police Service (IPS) is the principal policing agency in India, the main responsibility of policing lies with the state police and union territory agencies. The Indian Federal Government appoints and trains all IPS officers (senior and high-level police administrators), but most of the police personnel are recruited and trained by state agencies [17]. The Indian police structure is based on a military hierarchical style, which includes rigid pay scales and promotion schedules and no guaranteed leave [18]. The Home Ministry has financial, organisational, and political control at the federal and state levels [4]. The public image of the police tends to be poor, with suggestions of corruption and a perception that the police protect governments rather than individual citizens [16,19]. With its great diversity, colonial past, and political challenges, policing in India is demanding. The police tend to be understaffed, working long hours, and their relationships with the public are often fractious [19]. Constables make up much of the workforce, but their pay is low. 

Mental health law in India was institutionalised by the British with the introduction of the lunacy acts in 1858. This was replaced in 1912 by the Indian Lunacy Act of 1912—both were modelled on British laws and reflected the attitudes towards people with mental health illness at the time. The laws included a focus on containment and a bio-medical treatment model based on Western values [20]. Suicide was a criminal offence and only decriminalised recently in India, in 2018. The new mental health legislation, the Mental Healthcare Act, 2017 [21], which commenced on 29 May 2018. The 2017 Act states that “any person who attempts to commit suicide shall be presumed, unless proved otherwise, to have severe stress and shall not be tried and punished” (Section 115(1)). Police officers are expected to play an important role in relation to people who have self-harmed or attempted suicide in a range of ways, including taking a person believed to have a mental illness or be a risk to themselves into protection [21] S100 and S306. The act also highlights that “the appropriate Government officials including police officers and other officers of the appropriate Government are given periodic sensitisation and awareness training on the issues under this Act”. (Chapter 4, p. 15). The role of the police in terms of suicide and self-harm prevention and dealing with families and communities has been highlighted in some countries (e.g., the UK), but implementation can be challenging [22].

### 2.2. Sampling and Recruitment

Sampling was purposive, and we aimed to select participants from a range of ranks to gain insight into strategic and practise experiences and thinking. There is a smaller sampling pool for the higher ranks. To ensure confidentiality, we have categorised Inspector Generals of Police, Superintendents, Deputy Commissioners of Police as high-ranking, Head Constables, Circle Inspectors, Sub Inspectors, and Assistance Sub Inspectors as mid-ranking, and Constables as low-ranking.

Participants were identified through the Rajasthan Police Academy on behalf of the research team. The academy created a list of potential participants and sent bilingual information leaflets and consent forms (English and Hindi) by email. Interested individuals returned the signed consent form directly to the local contact (second author), and an interview was arranged at a convenient time. Participants were interviewed by the second and third authors in Hindi, the principal language of Rajasthan. Interviews were conducted by telephone or Zoom, and all were recorded with participants’ permission. We conducted 20 interviews with police personnel (see Table 1). 

### 2.3. Data Collection and Analysis

A semi-structured interview guide was used, focusing on questions about experiences with suicide and self-harm, challenges in responding to suicide and self-harm, and training and support needs. The guide was developed based on a literature review [3,8,14,22,23], the research question, and our interest in the topic. The second and third authors especially highlighted the importance of considering suicide within the police force. Interviews were conducted by the second and third authors in Hindi and lasted between 30–90 min. Recordings were transcribed, anonymised, and translated as soon as possible. Transcribed data was analysed using a reflective thematic approach [24] in NVivo 12 (a qualitative data analysis package). We used a critical realism ontology as it acknowledges different perspectives and interpretations of reality that are influenced by language, culture, and social structures. Critical realism allowed us to focus on the lived experiences of participants while also considering how some of the socio-cultural and policy aspects constrain and afford opportunities to participants [25]. This includes taboos and stigma around self-harm and recent changes in the legislation surrounding self-harm and mental health. Reflective thematic analysis is an interpretive approach focused on capturing a “core idea or meaning (what is shared and unites the observations in the theme is meaning), and the telling of an interpretative story about it” [26] (p. 2). Codes are constructed by the researchers interacting with the data and bringing their own experiences and backgrounds to the analysis process. The approach is suited to research aiming to understand people’s experiences and perceptions of a particular phenomenon in the contexts in which they occur [24].

The first author read and reread interview transcripts to develop themes that captured patterns and complexities. Initial codes were constructed, discussed with the second and third authors, and revisited iteratively, identifying semantic and latent codes. We used the concepts of organisational and operational factors as introduced by Hart and colleagues [8] to inform the categorisation of reasons given for suicide. Codes were clustered to find the final themes. Data were analysed within and across transcripts to identify patterns and check for inconsistencies and unusual experiences. The final codes were developed in consultation with the second and third authors. Findings were taken back to senior staff in the Rajasthan Police Academy for reflections and elaborations of the findings, a practise called Member Reflections by Tracey [27]. Members in this context are members of the police with experience in the field and as educators and trainers. This ensured that the research was more meaningful and had practical application.

To ensure anonymity, data extractions are only tagged with participants’ rank (high, mid, or low) and gender. Omitted data is represented by “[…]”. The study had ethical approval from the University of Manchester Ethics Committee. The Rajasthan Police Academy also reviewed and approved the research study.

## 3. Findings

We identified three main interlinking themes.

### 3.1. “It’s Such a Stressful Job”—The Police Organisational Environment Taking Its Toll

Participants made sense of suicide and self-harm in the police by highlighting the challenges encountered in a police job. However, participants moved on to emphasise how these challenges linked to challenges experienced in people’s personal lives. Over time, this can lead to stress and hopelessness, resulting in police staff killing themselves. We have categorised work-related challenges into occupational, organisational, and individual factors (informed by [11]). Organisational challenges were mentioned overwhelmingly as the main causes of stress; in terms of occupational challenges, a high workload was the most important issue.

Table 2 provides a list of challenges mentioned by participants. Similar to previous work, participants mentioned organisational factors far more frequently than occupational challenges. It has been suggested that police can deal with occupational challenges better as these are “part of the job” and can be expected, e.g., [3,11].

Participants tended to talk about the suicide of police staff in relation to high levels of stress; some called it mental stress. The following quote illustrates the link between work stress and occupational and organisational factors. The participant was talking about stress as a reason for suicide in the police:

*Very important part, I would say experience, during my eight years of posting, is, because it is such a stressful job because you don’t have fixed hours of working, and because there is shortage of manpower, the manpower we have is overburdened with work.*

*(High-ranking, female)*

The following quote highlights the importance of working conditions and working relationships:

*Self-harm in police organizations is mostly on the lines where the working conditions are not good, where the professional relations between the subordinate and the superior is not good.*

*(Low-ranking, male)*

Some participants specially highlighted tensions between senior and lower ranks and also tensions between different groups within a department, as can be seen in the following two quotes: “Police officers should treat lower staff just like human being not like machines. Police officers should change their attitude towards lower staff.” (High-ranking, male) and:

*The revengeful attitude of the seniors works like anything in Police Department Sir because transfer and backbiting are the biggest issues. There are several groups within the organization who connives against one another, and as a result, some people get transferred and there starts the concept of revenge, not in terms of fighting. Many are unable to bear this stress.*

*(Low-ranking, male)*

Occupational challenges when working with victims and their families and communities were not mentioned by participants when trying to explain why police staff may kill themselves; the following quote is an exception:

*Here are lot of situations where I’ve encountered 3–5 suicides committed by the family, by the friends or by the lovers, but that really create huge mental mark on the psyche of the police officer, too.*

*(High-ranking, male)*

Generally, the work with victims and their families might be described as difficult at times, but not as something that leads police personnel to kill themselves. Most participants talked about suicide as a result of organisational challenges having an impact on people’s personal lives, as the following quote illustrates:

*In the police force, working conditions are poor. They have long working hours, have difficulty in getting leave when they need them, their families have expectations that they are unable to meet. Their pay is very little. They don’t spend much time with their families.*

*(high-ranking, female)*

Job demands such as working away from home can leave staff feeling lonely or cut off from their family and also result in conflict and tension, which in turn can lead to suicidal actions:

*In my view family factors plays a definite role in cases of self-harm. Few months back an IAS [Indian Administrative Service] officer did suicide in front of train and the reason was familial. I can’t say that reasons are never professional but in most of the cases reasons are related to family and mostly due to lack of understanding.*

*(Mid-ranking, female)*

What these quotes show is that negative occupational and organisational factors can negatively impact family relationships over time.

### 3.2. ‘You Have to Be Mentally Strong’—How to Cope with the Police Role

Participants seemed to try and distance themselves from the idea that police staff who commit suicide are weak or unable to cope. As shown above, the suicide of police staff was generally framed as a result of work and organisational factors impacting family and community relationships. This came across as an understandable outcome due to external pressures beyond the person’s control.

All participants indicated that society would see suicide as a sign of weakness, not being able to cope with the stresses and strains of life and work:

*People who commit such episodes of self-harm, society consider them as weak. Society does not justify them. People think what is the need of suicide, he must be-a weak person. He has to manage all the situations but what is the need of suicide. He is not a fighter. Society thinks that he is a third-class person. He is a weak person, can’t afford stress.*

*(Mid-ranking, male)*

Very few phrased it to indicate that they personally agreed with this point of view. As mentioned earlier, most participants talked about suicide in terms of stress or mental distress but far less in terms of mental illness. The following quote is an exception, referring to people with existing mental health conditions who enter the police and then struggle with the requirements of the role:

*Psychiatric cases are another challenge with the uniform forces. People who are psychiatric cases do enrol in police forces and get into the permanent job after initial two to three years.*

*(High-ranking, male)*

Rather than talking about weakness in relation to police staff, the need to be strong was highlighted as an essential characteristic for the police role:

*Somebody who’s mentally strong is needed in this profession because if you are not mentally strong then that is the biggest self-harm that you can do to yourself.*

*(High-ranking, female)*

Research in Western countries suggests that the police culture is one of masculinity, independence, and emotional control, where it is difficult to ask for help [28]. Equally, Thakre and colleagues [29] outline how Indian police staff are expected to be tough, brave, and resilient, and that mental health illness is seen as a weakness.

Alcohol and drugs were framed as coping mechanisms, albeit destructive ones:

*… because it is such a stressful job because you don’t have fixed hours of working, and because there is shortage of manpower […] so, the tendency to getting addicted to liquor or drugs is very common in police force.*

*(High-ranking, female)*

Longer-term use of alcohol and drugs was linked to domestic abuse and debts by several participants:

*In my view, police personnel due to excessive stress end up taking alcohol as a way of relief, this then leads to arguments in the family and domestic abuse.*

*(High-ranking, female)*

This indicates that the long-term requirement for strength negatively impacts their ability to cope or ask for help. Although it has been suggested that people entering the police force are more likely to be mentally robust and well-prepared in comparison to the general population [3], a recent systematic review concluded that police personnel have higher levels of depression (14.6% vs. 3.8%), suicidal ideation (9.2% vs. 3.3%), and hazardous drinking (25.7% vs. 16.6%) compared to the general UK population [30].

### 3.3. ‘Police Demands a Certain Image’—Help-Seeking Opportunities Need to Be Flexible

The importance of mental strength and the ability to cope when working in the police force means that police staff are less likely to ask for help. The police role comes with certain values and expectations. Participants suggested that if a member of the police is unable to uphold this image, linked to perceptions of losing respect, it may lead to suicide:

*People bound in their social image and then something happened they thought that my social image will drop down and what people think about me. The major stress is at this time that what people think about me, what will society think about me?*

*(Mid-ranking, male)*

In a police context, challenges to one’s image tend to be linked to experiences of allegations and complaints:

*In some cases, people are very image conscious and that lead to mental stress also. Allegations are very common in police job. […] This leads to mental stress and ultimately suicide.*

*(Mid-ranking, male)*

Seeking help is generally seen as shameful and indicates that one is unable to cope:

*The person with suicidal tendency is also mentally weak as compared to a normal person. In our Indian society if any person is suffering from mental stress or I can say depression, and he went to the psychiatrist for help then the colleagues or other persons say that he must be mad, mental or fool that’s why he is going to psychiatrist. This is also a big social taboo in society.*

*(Mid-ranking, female)*

This is even more so in the case of the police:

*If this event [self-harm] has happened within the department, people brand that person as nobody should talk to him. It is like tarnishing his image further. Rather, there are chances that he will go further depressed.*

*(High-ranking, male)*

Acts of repeated self-harm are linked to suicide [31]. However, as one of the participants pointed out, it is unlikely that people will report self-harm in the workplace:

*Yes, we do come across self-harm and suicides among […] police staff […]. But the harm is hardly reported means if somebody is harmed and it doesn’t lead to suicide, very rarely it is reported.*

*(High-ranking, male)*

Seven participants talked about someone they knew in the police force who killed themselves. They portrayed this as a shock, again highlighting how unlikely it is for staff to ask for help or share their difficulties with others:

*Sometimes there is no obvious reason for suicide so we have to search reason. This is such a tragedy that many a times we cannot believe on it. One such incidence happened to me when one of my colleague committed suicide at the age of 54.*

*(Mid-ranking, male)*

Only a few participants highlighted the need to address negative attitudes towards suicide and mental health within the police force:

*People who join the police force are after all belong to the same community, it’s not that they have arrived from different planet. In that people who join police force held views and prejudices that they carry on holding which is a big challenge for us.*

*(High-ranking, female)*

The negative influence of British rule and colonialism on current policing was only mentioned by two participants. In particular, they saw this working climate creating a negative impact on staff relationships and the ability to ask for help and support: “Just like British Rule, ‘yes sir’ ‘no sir’ type of things are still prevailing here.” (Low-ranking, male)

In addition, these colonial rules were also described as responsible for some of the poor working conditions:

*We can’t make a union from constable to sub- inspector level. We don’t get childcare leaves in police department. Even government announced that but we cannot take leaves in police department. Our police department run on British rule.*

*(Mid-ranking, female)*

The Indian police largely maintained the colonial structures and rules. As mentioned earlier, these hierarchical, rigid structures restrict promotion schedules and leave and have resulted in poor pay for lower ranks [18]. A need to address challenging working conditions and, in particular, the level of workload and ability to take leave were strongly highlighted. Evidence from India suggests that police personnel may not ask for help for fear of job sanctions, stigmatisation, and missed job opportunities [32]. Indeed, feedback from participants indicated that opportunities to discuss issues are not taken up. The following quote is about a helpline for police staff that does not allow the caller to remain anonymous:

*They are still hesitant in calling. From a force of around 2500 men personnel under me, I would be receiving one call in 20 days, which is very less, I think. They should call more, but that system of breaking the hierarchy or comfort level maybe is not there.*

*(High-ranking, female)*

Some participants mentioned existing independent support options, and several highlighted the need to offer support that maintains confidentiality and anonymity:

*You will have to open and come out. It should be like you don’t have to disclose your name […] so it has to be the anonymous thing. Confidential anonymous because we have to address the shame first.*

*(High-ranking, female)*

We asked participants if they were aware that suicide had been decriminalised, and half indicated that they had been unaware. This is of concern as it may contribute to staff’s reluctance to disclose difficulties and ask for help. Unfortunately, none of the participants talked further about what influence the interpretation of the law may have on police personnel. Previous research has shown that where suicide is criminalised, people are unlikely to seek help, and the act is more likely to be stigmatised [31,33].

In addition to anonymous helplines and support, training on mental health issues and how to look after one’s own psychological well-being and a better understanding of self-harm and suicide were requested. The importance of good communication and listening skills for police personnel was highlighted as being beneficial, not only when working with the general public but also when dealing with colleagues, and in particular for police in leadership roles.

## 4. Discussion

This qualitative study shows how police personnel make sense of suicide in the police force based on negative interactions between the work environment, personal factors, and the family context. Findings are in line with other research, showing that organisational factors play a major role as well as high workload, an occupational factor [9,34]. Lack of leave, another factor highlighted here and in other studies with Indian police, is rarely considered in research in other countries. Research in Western countries has highlighted that mandatory holiday shifts, which prevent police personnel from participating in celebrations with family and friends, have a negative impact on staff well-being and family relationships [35]. Examples of the physical and psychological effects associated with overwork in the police force in China and the US include, amongst others, cardiovascular disease, diabetes, hypertension, depression, anxiety, and suicide [36]. These findings, together with the findings from the present study, underscore the importance of providing sufficient and timely leave. Positive steps are being taken in some Indian states, with an increase in the number of leave days in a year [37].

Suicide is described as more likely due to external pressures building up over time. Working in the police is linked to a specific image and expectations, which do not allow for weaknesses and inhibit help-seeking. This may be coupled with stigma attached to mental illness and suicide. A recent study by Weiss and Parkar [38] in India with people who had self-harmed or tried to kill themselves highlighted the importance of focusing on self-harm and suicide prevention rather than mental health. A mental health illness label was described as more stigmatising than self-harm or an attempt to complete suicide. They concluded that efforts to encourage help seeking should frame reasons for suicide and self-harm as based on social and structural features rather than mental health illness, which is what participants in the current study have focused on. These findings underscore the importance of considering cultural and local contexts. 

The historical and colonial context plays a very important role here, as it shaped the development and set-up of the police force as well as the understanding and treatment of mental ill health [39,40]. In addition to colonial values, social values have influenced policing structures and resulted in a patriarchal, hierarchical, and authoritarian work force. Positive organisational culture, climate, and leadership are invaluable [34]. Nuttman-Shwartz and colleagues [41] originally showed, in a random sample of the Israeli population, that how an individual views suicide is influenced by their perceptions of how the public views and judges suicide. In other words, how suicide is framed and talked about by the wider population impacts an individual considering suicide and/or help-seeking and how completed suicides are talked about. Similarly, research with the Australian police force has shown that just the perception that help-seeking indicates weakness or might interfere with job prospects can prohibit help-seeking. This is the case even when organisations aim to provide mental health support [42]. Perceptions around suicide need to be addressed at the policy and welfare levels, and the recently published Indian suicide strategy is a step forward [43].

Recent developments provide major opportunities for India with the introduction of the Mental Health Act 2017 [21], which puts a focus on patients and their families and aligns with the principles of human rights as enunciated in the United Nations Convention on Persons with Disabilities (UNCRPD) and the National Suicide Prevention Strategy [43], which focuses on reducing the stigma around suicide and working with key stakeholders. Our study suggests that how suicide and self-harm are experienced and talked about in the police force has important implications not only for suicide prevention and interventions at the force level. It can inform the gatekeeping role of police in suicide prevention in the general public. This is essential, as the Indian Mental Health Act 2017 [21] expects the police to take a more active role.

Based on their systematic review of initiatives in the Asia-Pacific region, Pothakool and Meethiam [44] recommend that “police agencies develop training and engage in collaborative engagement with a range of health and community stakeholders to evolve police officers’ views towards a public health perspective in relation to policing activities” (p. s23). They argue that this would allow to address issues such as mental ill-health, suicide, and harm more effectively. These activities would need to be underpinned by community policing principles, trust between the police and the community, and sufficient resources.

The current study has some limitations. It is a small qualitative study focusing on one Indian state. However, we have managed to recruit participants from a hard-to-reach and busy professional group whose opinions and behaviours can have a significant impact on suicide prevention, not only in the force but also in the general public. Our study starts to address the need for a more in-depth understanding of the challenges police officers experience in South Asia. Suicide and mental health in the police force are very sensitive topics and a qualitative approach is most appropriate. Further research with other key stakeholders, such as prison officers and families of police personnel, would be helpful in informing suicide prevention.

Our findings have implications for practise and policy. Importantly, they highlight that action needs to move beyond an individual focus to address organizational, institutional, and societal factors. Increased suicide and mental health literacy will be helpful in improving attitudes, skills, and knowledge when dealing with one’s own and colleagues. It will also increase confidence and skills when dealing with the public. However, approaches need to take the local and country-wide context into account and not only focus on the police force but also address perceptions around suicide in the wider population. Improvements in working conditions and organisational structures need to be addressed at the state and country levels.

## Figures and Tables

**Table 1:** Summary of participants demographics.

Demographic Information (*n* = 20)	
**Age**	Range 28–57 Mean 39 years
**Group**	High-ranking (5)
	- 3 females - 2 males
	Mid-ranking (8)
	- 6 females - 2 males
	Low-ranking (7)
	- 2 females - 5 males
**Length in current post**	7 months to 7 years

Not all participants were asked their religion; those that were identified themselves as Hindu (*n* = 14).

**Table 2:** Challenges experienced by police staff.

**Occupational Challenges**
Distressing work experiences impacting on mental health
Not enough sleep
Stressful job with lots of pressures
Over-burdened with work
**Organisational challenges**
Recruiting over-qualified people as constable who are then dissatisfied
Poor pay structure
Grievances not dealt with appropriately
Humiliation by other officers
Urgent requests for leave not granted
Shortage of manpower resulting in lots of overtime
Lack of resources
Allegations against officers
Postings far away from family
Work environment not being supportive
Lack of support and interest by higher officers
No union on police up to inspector level
**Individual challenges in a family context**
Debt and poverty
Societal changes–e.g., change from extended families to nuclear families; more individualistic focus
Relationship issues and family reasons (e.g., attraction to a colleague, being away from home a lot, not able to share childcare)
Women experiencing domestic violence
Drug or alcohol addiction

## Data Availability

Data sharing is not applicable due to ethical concerns (participants may be identified).
